# Protease inhibition, *in vitro* antibacterial and IFD/MM-GBSA studies of ciprofloxacin-based acetanilides

**DOI:** 10.1371/journal.pone.0281044

**Published:** 2023-03-31

**Authors:** Rabia Akhtar, Ameer Fawad Zahoor, Asim Mansha, Shagufta Kamal, Samreen Gul Khan, Zohaib Raza, Kulsoom Ghulam Ali

**Affiliations:** 1 Department of Chemistry, Government College University Faisalabad, Faisalabad, Pakistan; 2 Department of Chemistry, The Superior University Lahore, Faisalabad-Campus, Faisalabad, Pakistan; 3 Department of Biochemistry, Government College University Faisalabad, Faisalabad, Pakistan; 4 Department of Pharmacology, Government College University Faisalabad, Faisalabad, Pakistan; Aligarh Muslim University, INDIA

## Abstract

In this study, we have investigated ciprofloxacin-based acetanilides for their *in*-*vitro* inhibitory study against gram +ve, -ve bacteria and serine protease activity. The compounds **4e** and **4g** showed excellent antibacterial activity against *Bacillus subtilis* with a zone of inhibition (ZI) values of 40 ± 0.9 mm, 37 ± 1.4 mm and with MIC values of 4.0 ± 0.78 mg/mL, 3.0 ± 0.98 mg/ML respectively, while **4a** and **4i** were found most active against *Escherichia coli*, with ZI values 38 ± 0.1 mm, 46 ± 1.8 mm and with MIC values of 1.0 ± 0.25 mg/mL, 1.0 ± 0.23 mg/mL respectively. All derivatives (**4a-j**) significantly inhibited the catalytic activity of serine protease, while **4a** exhibited a maximum (100%) inhibitory effect at 96 minutes having 22.50 minutes t12, and non-competitive inhibition with 0.1±0.00μM *K*_*i*_. The IFD/MM-GBSA studies highlighted the binding mode of **4a** for protease inhibition and indicated improved binding affinity with –107.62 kcal/mol of ΔG_bind_.

## Introduction

**Fig 1:**
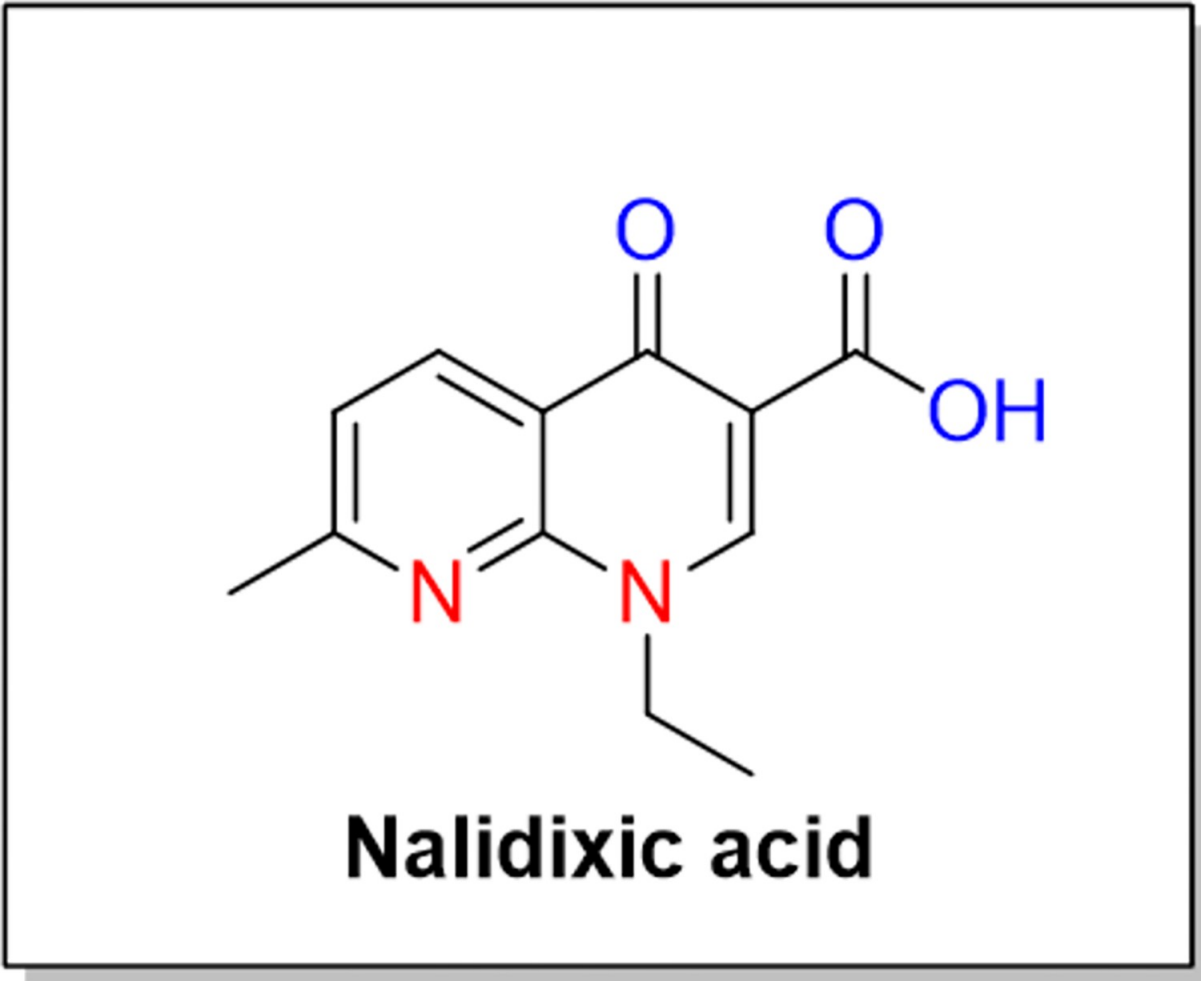
Structure of nalidixic acid.

A new era of synthetic quinolone antibacterial agents started in 1962 with the discovery of nalidixic (a prototype of quinolone) by George Lesher as a byproduct during the commercial preparation of chloroquine (an antimalarial) (Fig 1). Nalidixic acid on oral administration is rapidly absorbed and converted into active hydroxyl nalidixic acid by liver, mostly excreted by the kidneys act there as bactericidal. It is used for the treatment of urinary tract infections caused by Gram-negative bacteria. However, its selectivity only for Gram-negative bacteria, photosensitivity reactions, as well as the convulsive tendency in seizure disorders patients, limited its extensive use [1–6].

Fluoroquinolones constitute a group of quinolones having fluorine substituent at position 6 of their bicyclic rings, which has led to the development of a marvelous group of most frequently used synthetic antibiotics as well as protease inhibitors [7, 8]. Ciprofloxacin is one of them which was first patented in 1983 and approved for clinical use in 1987 and showed excellent potency against Gram-negative bacteria [9]. Later, other fluoroquinolones, such as levofloxacin, gatifloxacin, moxifloxacin, and gemifloxacin were developed with better activity against Gram-positive bacteria. They are currently the most commonly prescribed antibacterial agents in the world for the treatment of a wide variety of bacterial infections in human beings as well as in animal husbandry.

**Fig 2:**
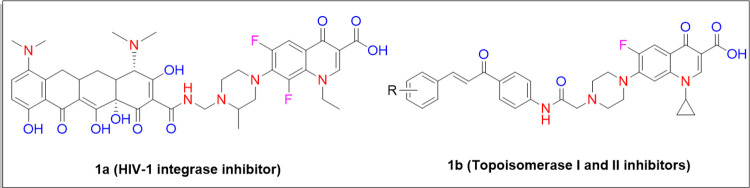
Fluoroquinolones as HIV-1 integrase, topoisomerase I and II inhibitors.

They have been widely used to inhibit HIV-1 integrase enzyme [10] and also act as DNA topoisomerases I and II inhibitors [11] (Fig 2). A large number of pharmaceutical chemists are involved in structural modifications of the core fluoroquinolone to develop new potent antitumor, anti-HIV, as well as antimycobacterial agents with a broader spectrum, improved efficacy and less chance of developing resistance. Apart from the above applications, anti-inflammatory [12] and antifungal [13] properties have also been exhibited by these motifs [14–16].

Resistance to existing pharmaceutical agents is a major health problem that can be combated by creating a new generation of antibiotics having a different mechanism of action. As a part of our study attempting to further optimize the fluoroquinolones’ antibacterial activity, we have investigated the effect of *N*-4-piperazinyl ciprofloxacin-aniline derivatives [17] on *Bacillus subtilis* and *Escherichia coli*. The results revealed that the introduction of different substituents at phenyl ring significantly influenced their antibacterial activity against the targeted bacterial cultures. Moreover, we evaluated the serine protease inhibition potential of the designed ciprofloxacin derivatives with significant results. As per our knowledge, no study before this work involved the evaluation of *in vitro* inhibitory effect of ciprofloxacin derivatives against serine protease.

## Materials and methods

### Chemistry

The synthesis of *N*-4-piperazinyl ciprofloxacin derivatives **4a-j** was carried out by refluxing ciprofloxacin hydrochloride in methanol using a catalytic amount of sulfuric acid, resultantly carboxylic acid moiety was converted into methyl ester **2**. 1.45 mM of this ester (**2**) were treated with a series of substituted anilides **3a-j** (2.17 mM) in the presence of pyridine (9 mM) and dichloromethane (20 mL) to obtain targeted compounds within 72–96 h. *N*-hexane was used in the workup procedure to get precipitates which were purified by recrystallization/column chromatographic techniques [17].

### Antibacterial activity

Antibacterial activity of ciprofloxacin derivatives was evaluated by the disc diffusion method as reported earlier against *Bacillus subtilis* and *Escherichia coli* [18]. 100 μL of suspension made up of 10^8^ colony-forming units of bacteria was spread on nutrient agar media. A compound solution having 5mg/100μL concentration in DMSO was imbedded on discs while ciprofloxacin as a positive control was carefully deposited in the discs on the agar culture plates (already immunized with bacterial cells). Later cultured plates were incubated at 4°C for 1 hour followed by 37°C for 24 hours. All the results were compared with the control by calculating zone of inhibition (ZI) values of bacterial growth as an average diameter around the discs in mm.

### Minimum inhibitory concentration (MIC)

Resazurin microtiter-plate assay was used to determine the minimum inhibitory concentration (MIC) values of all the compounds against the same microorganism. To fill the well plates, 10 mg/mL solution of standard antibacterial drug in 10% DMSO, 100 μL solution of compounds (made up by mixing 100 mg/mL (w/v) concentration of compounds in 10% DMSO), and nutrient broth were used. A multichannel pipette was employed to dilute the solutions up to two folds (50 μL of compounds in descending concentrations in each plate). The next step was the preparation of the resazurin indicator (resazurin tablets (270 mg) + 40 mL distilled water), of which 10 μL quantity was poured into each well along with 30 μL iso-sensitized broth. 10 μL of bacterial suspension was transferred into each well to attain a 5 x 10^5^ cfu/mL concentration. Each plate (containing positive and negative controls) was incubated at 37°C for 24 h followed by 25°C for 48 h.

### Determination of inhibitory rate of serine protease

500 μL of purified serine protease solution (indigenously isolated from *B*. *subtilis* sp.) was incubated at 37°C for 30 minutes with 950 μL of 2% casein solution (10 mM tris-HCl at pH 8.0) and 500 μL of the synthesized compounds. The reaction was terminated by adding 10% trichloroacetic acid (TCA) and the Optical Density (O.D.) was monitored at λ_600_ in UV-Vis spectrophotometer (UV-1800, Shimadzu, Kyoto, Japan).


$$Inhibitory\;rate\;of\;protease\;(\%)=(100\times (V_{0}–V_{n})/V_{0})$$ (1)

Where,

*V*_n_ = Rate of reaction

*V*_0_ = Rate of casein free-protease reaction system (control)

### *In-silico* molecular modelling

The binding of compounds was further simulated by *in-silico* Induced Fit docking and Prime/MM-GBSA protocol in Schrӧdinger 2018–4 molecular modelling software using Maestro 11.8 interface [19].

#### a. Ligands preparation

The three-dimensional (3D) conformer of ciprofloxacin (CID: 2764) was retrieved from the PubChem database. The synthetic compound **4a** was sketched in Maestro 11.8, and low-energy 3D conformations were produced in LigPrep module using OPLS 2005 forcefield. The tautomeric and ionization states were generated using EpiK at 7.0 ± 2.0 pH. While stereoisomeric chirality was retained in original state, and up to 32 conformations were generated for each ligand.

#### b. Protein preparation

The X-ray crystalized 3D structure of serine protease (PDB ID: 3TT7, 2.56 Å resolution) was retrieved from RSCB Protein Data Bank (http://rscb.org/). The macromolecule was prepared in Protein Preparation Wizard using OPLS 2005 forcefield. The structure was preprocessed to assign bond orders, zero-order bond to metals, disulfide bridging, protonation, removal of water molecules beyond 5 Å of HET group, and HET states were generated using Epik at 7.0 ± 2.0 pH. The macromolecule structure was optimized for H-bonds assignment by sampling water orientations with PROPKA at 7.0 pH. The restrained minimization was performed to set convergence of heavy atoms up to 3 Å.

#### c. Induced fit docking

Standard IFD protocol was followed to produce 20 poses for each ligand. The binding site was specified to the centroid of co-crystalized ligand. Initially, Rigid Receptor Glide Docking (RRD) of each ligand was performed, using softened van der waals radii scaling <0.5, to produce 20 conformations with Coulomb-vdW <100 and H-bond score <-0.05. These RRD complexes were further processed in the Prime refinement, and induced fit receptor structure was produced by conformational sampling and optimization of side chains of receptor’ residues within 5 Å of docked-ligand conformation. The Prime refinement was followed by Extra-Precision (XP) Glide re-docking of ligands into induced fit receptor’ conformations within 30 kcal/mol energy of lowest energy structure, and poses (or IFD complex) were scored/ranked by combination of Prime energy and Glide XP scoring functions.

#### d. Prime/MM-GBSA free energy estimation

The IFD complex was further employed in Prime/Molecular Mechanics Generalized Born Surface Area (Prime/MM-GBSA) simulation to calculate the accurate binding free energy (ΔG_bind_) using the following equation:

$$\Delta G_{bind}=\Delta E_{MM}+\Delta G_{solv}+\Delta G_{SA}$$ (2)

Where,

ΔE_MM_ = Difference in minimized IFD complex energy and Σ energies of unbounded receptor and ligand;

ΔG_solv_ = Difference in GBSA solvation energy of IFD complex and Σ solvation energies of unbounded receptor and ligand;

ΔG_SA_ = Difference in surface area energy of IFD complex and Σ surface area energies of unbounded receptor and ligand.

### Statistical tests

All the experiments were conducted in triplicates to obtain the mean value. To verify the experimental results, a statistical test (ANOVA) was accomplished by considering a probability value of 0.05.

## Results and discussion

### Chemistry

**Scheme 1:**
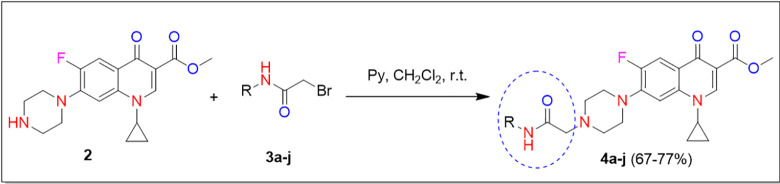
Synthesis of analogues of N-4-piperazinyl ciprofloxacin derivatives 4a-j.

The synthesis of *N*-4-piperazinyl ciprofloxacin derivatives **4a-j** has been demonstrated in the synthetic route as shown in Scheme 1 and Table 1 [17]. Esterification of ciprofloxacin was carried out by refluxing ciprofloxacin hydrochloride in methanol using a catalytic amount of sulfuric acid [20]. The resulting methyl ester **2** was treated with a series of variously substituted anilides **3a-j** (prepared by the reaction of the corresponding amine with bromoacetyl bromide [21–23]) in the presence of pyridine (as base) and dichloromethane (as solvent) to obtain the targeted compounds **4a-j** in good yields (67–77%) [17].

**Table 1:** Synthesized *N*-4-piperazinyl ciprofloxacin derivatives 4a-j [17].

Sr. No.	Compound	R	Yield (%)	Sr. No.	Compound	R	Yield (%)
1.	**4a**	C_6_H_5_	67	6.	**4f**	3-ClC_6_H_4_	71
2.	**4b**	2,4-Me_2_C_6_H_3_	68	7.	**4g**	2-FC_6_H_4_	75
3.	**4c**	2-MeOC_6_H_4_	72	8.	**4h**	4-FC_6_H_4_	77
4.	**4d**	RNH = O(CH_2_CH_2_)_2_N	70	9.	**4i**	3,4-Cl_2_C_6_H_3_	69
5.	**4e**	4-ClC_6_H_4_	68	10.	**4j**	2-ClC_6_H_4_	70

### Antibacterial activity

Antibacterial activity of the synthesized derivatives **4a-j** has been examined against drug-susceptible Gram-positive (*Bacillus subtilis*) and Gram-negative (*Escherichia coli*) bacteria. The zone of inhibition (ZI) and minimum inhibitory concentration (MIC) values against the aforementioned bacterial cultures have been presented in Table 2. The results were compared with standard drug ampicillin, ibuprofen and positive control ciprofloxacin. At a glance, most of the derivatives showed higher antibacterial values than the parent drug (ciprofloxacin). Compounds **4e** and **4g** exhibited excellent antibacterial activity against *Bacillus subtilis*, as compared to the standard drugs and positive control (ciprofloxacin) having ZI values 40 mm and 37 mm, respectively. While, results of compounds **4d**, **4h** and **4i** were in close range to unmodified ciprofloxacin. The antibacterial activity of compound **4j** was somewhat lower than that of the parent compound. In case of *Escherichia coli*, compounds **4a, 4e** and **4i** were the most active ones depicting zone of inhibition values 38 mm, 34 mm and 46 mm, respectively. However, compounds **4b**, **4f** and **4h** exhibited lower antibacterial activity in case of above mentioned bacteria as compared to the reference antibiotics, while compound **4c** remained inactive at all against *E*. *coli*.

**Table 2:** Zone of inhibition values (millimeters) of compounds 4a-j.

Compound	*Bacillus subtilis*		*Escherichia coli*	
	ZI (mm)	MIC (mg/mL)	ZI (mm)	MIC (mg/mL)
**4a**	31.5 ± 1.3	1.50 ± 0.6	38 ± 0.1	1.0 ± 0.25
**4b**	32.5 ± 0.6	1.0 ± 0.75	6.0 ± 0.0	1.0 ± 0.25
**4c**	29.75 ± 0.0	3.0 ± 0.25	ND	1.0 ± 0.75
**4d**	29 ± 1.25	1.50 ± 0.00	29 ± 1.3	2.0 ± 0.87
**4e**	40 ± 0.90	4.0 ± 0.78	34 ± 0.2	1.0 ± 0.02
**4f**	31 ± 0.4	1.0 ± 0.65	11 ± 0.3	1.0 ± 0.00
**4g**	37 ± 1.4	3.0 ± 0.98	14 ± 0.1	5.0 ± 0.35
**4h**	29 ± 1.2	1.0 ± 0.75	7.0 ± 0.1	1.0 ± 0.45
**4i**	29 ± 0.7	1.0 ± 0.89	46 ± 1.8	1.0 ± 0.23
**4j**	26 ± 0.1	5.0 ± 0.75	21 ± 1.2	1.0 ± 0.00
**Ampicillin**	18 ± 1.0	9.0 ± 1.1	12 ± 0.6	16 ± 1.1
**Ibuprofen**	15 ± 0.9	14.0 ± 1.0	11 ± 0.5	21 ± 1.4
**Ciprofloxacin**	29.3 ± 1.0	0.3 ± 0.0	31.1 ± 1.2	2.2 ± 0.1

MIC = Minimum inhibitory concentration; ZI = Zone of inhibition; ND = not detected.

### Structure-activity relationship (SAR)

**Fig 3:**
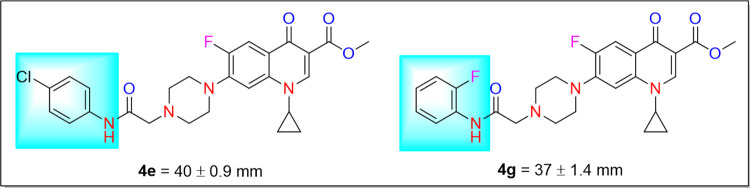
Structures of compounds 4e and 4g active against *Bacillus subtilis*.

The SAR analysis indicated the difference in the activity of these compounds because of their electron-donating and electron-withdrawing groups present at the anilide ring. It was observed that chloro and fluoro substituents at anilide ring imparted a vital role in enhancing the antibacterial activity against *Bacillus subtilis*. As two compounds, **4e** having electron-withdrawing chloro functionality at *para* position and **4g** having electron-withdrawing F functionality at *ortho* position of the anilide ring showed notable contribution towards the antibacterial activity of 40 ± 0.9 mm and 37 ± 1.4 mm, respectively (Fig 3).

**Fig 4:**
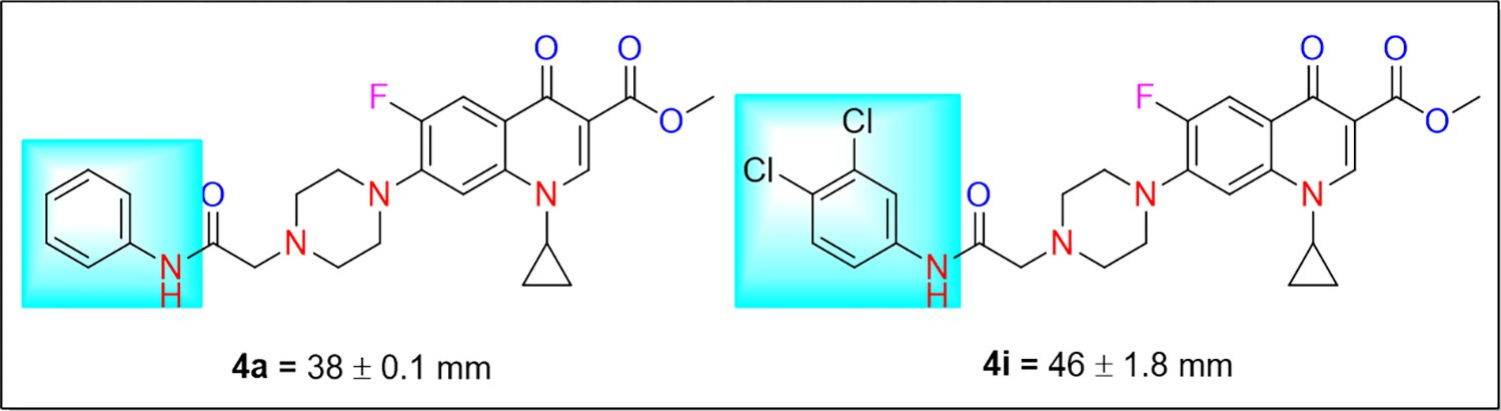
Structures of compounds 4a and 4i active against *Escherichia coli*.

However, an arbitrary behavior was observed in the case of *Escherichia coli*. It was found that **4e** having electron-withdrawing Cl group at 4- and **4i** having Cl group at 3,4-positions of the anilide ring exhibited higher activity as compared to **4f** with 3-Cl, **4g** with 2-F and **4h** with 4-F groups at anilide ring (Table 2 & Fig 4). Besides this, compound **4a** displayed promising activity against *Escherichia coli* with larger diameter of zone of inhibition (38 ± 0.1 mm). In conclusion, *in vitro* antibacterial study of the synthesized *N*-alkylated ciprofloxacin derivatives, **4a-j** revealed that these hybrid structures comprising electron-withdrawing groups at anilide moiety strongly inhibited the growth of *B*. *subtilis* as compared to *E*. *coli*.

### Effect of *N*-alkylated ciprofloxacin derivatives 4a-j on the activity of serine protease

**Fig 5:**
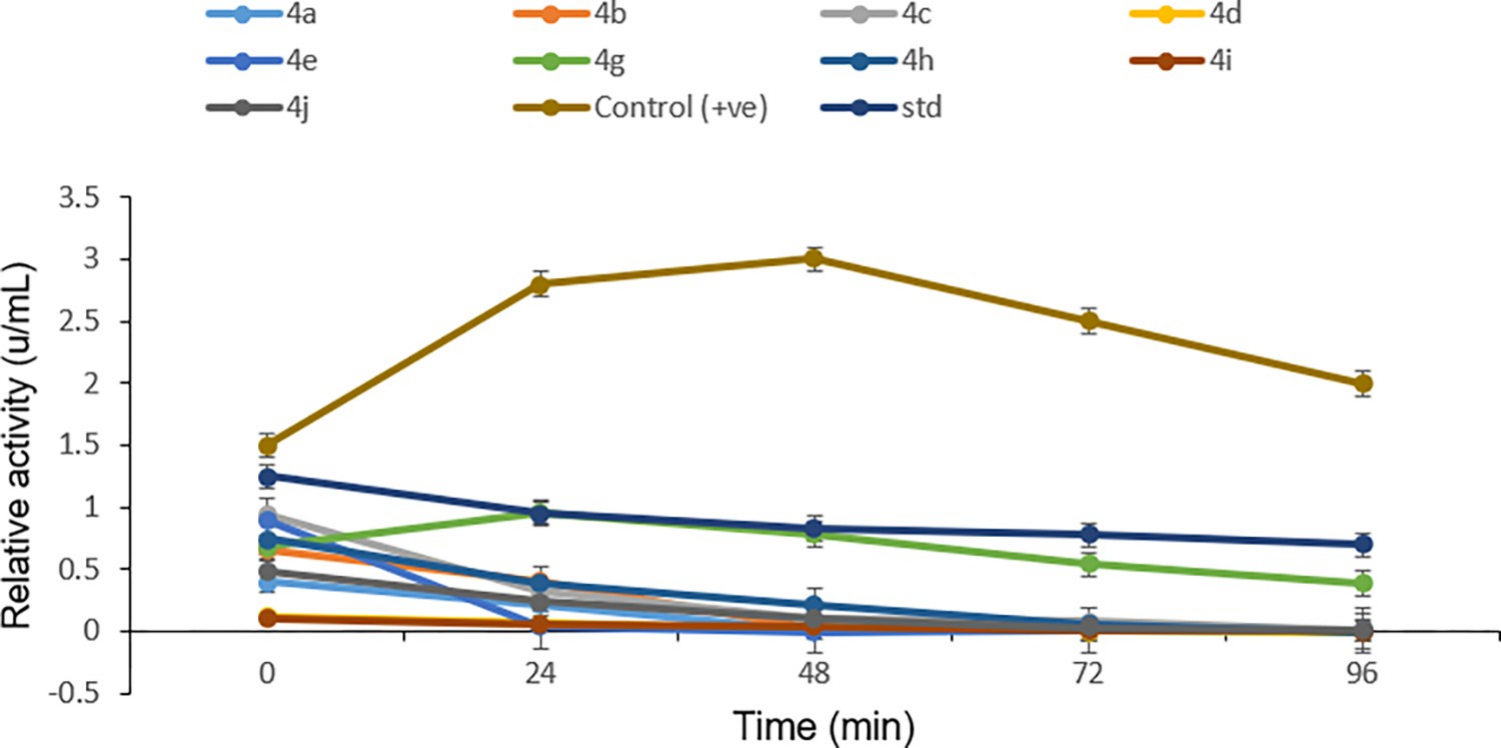
Influence of ciprofloxacin derivatives 4a-j on the activity of protease.

**Fig 6:**
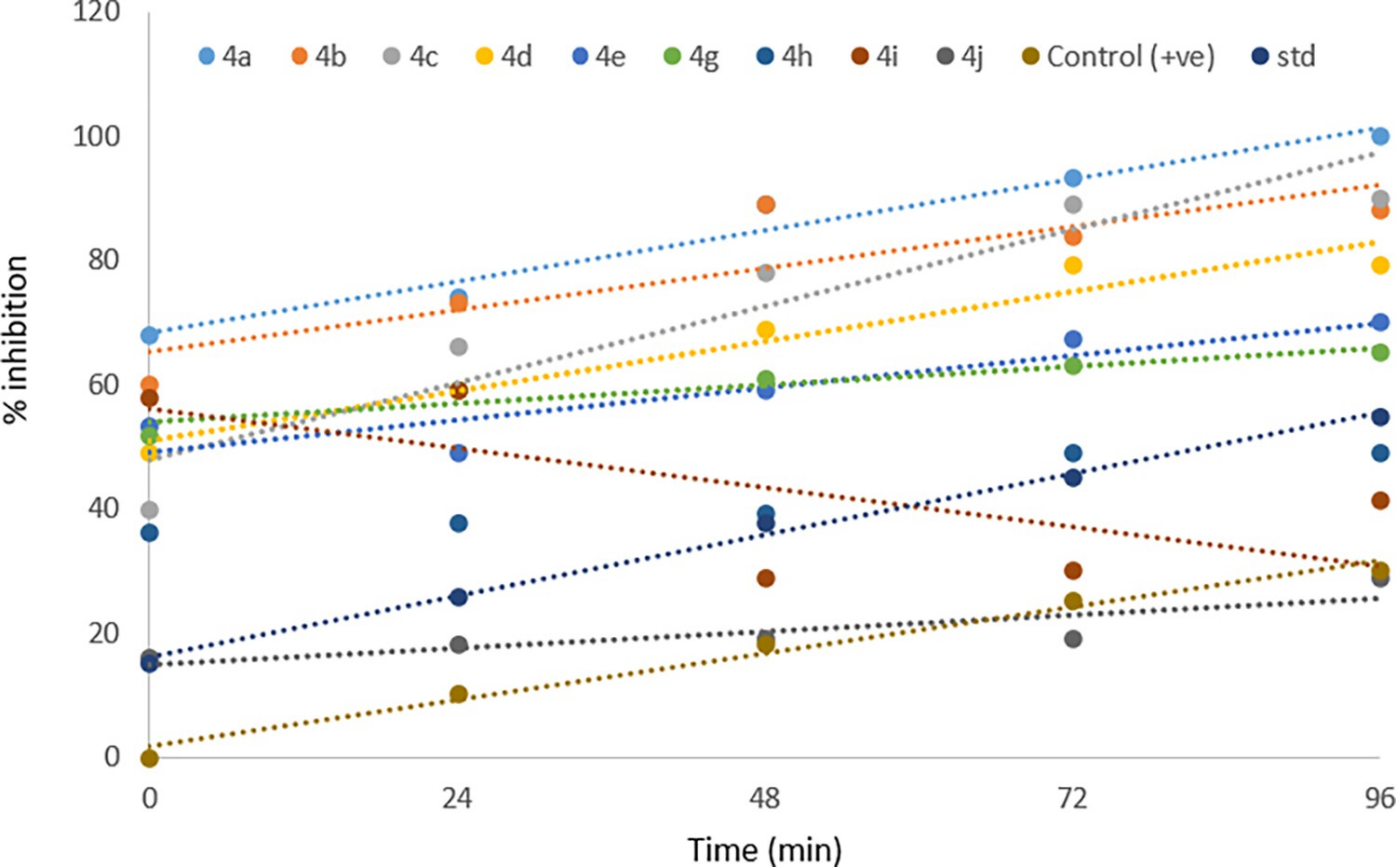
Determination of % inhibition of protease activity (μ/mL) 4a-j.

**Fig 7:**
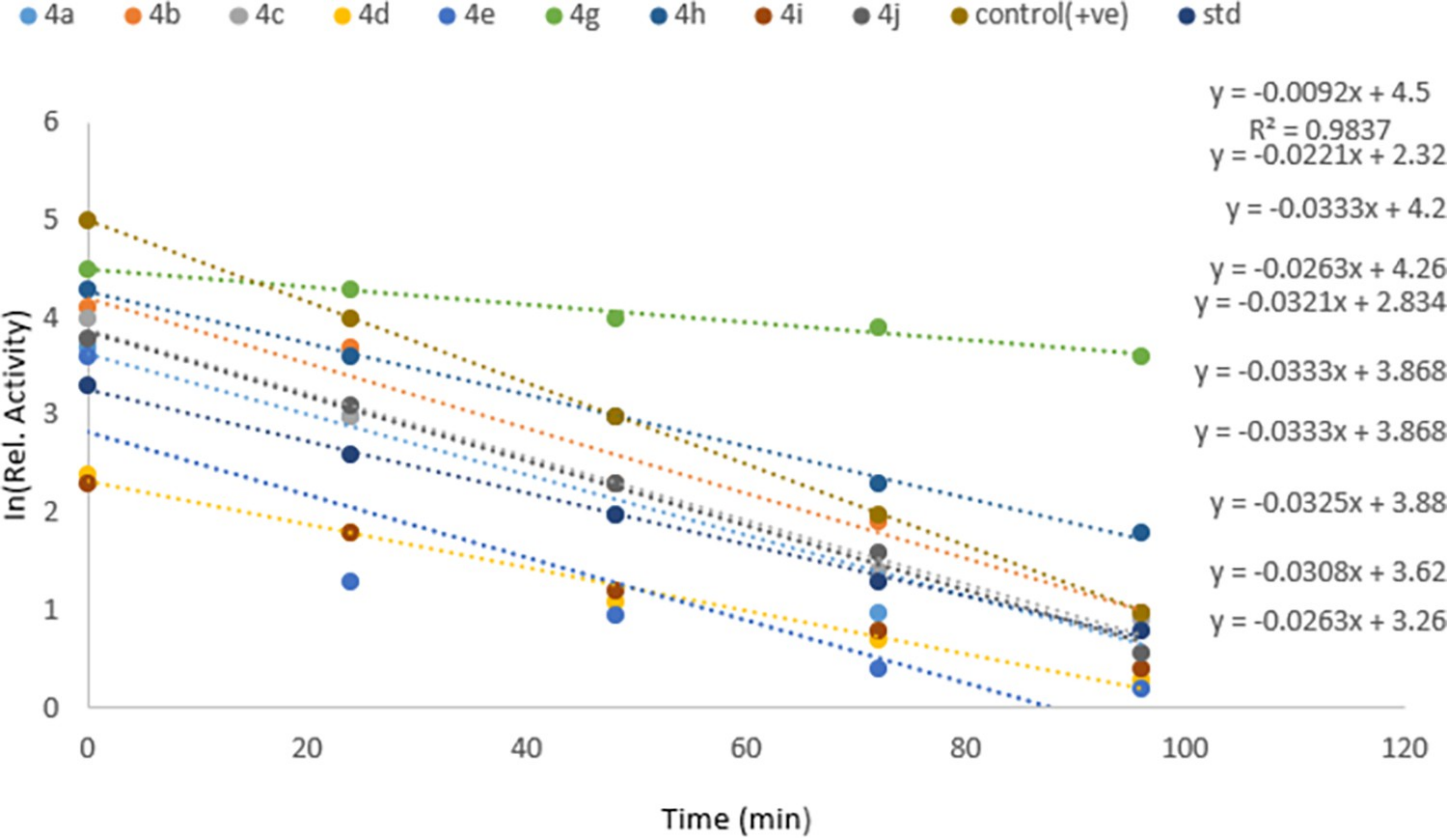
Determination of half-life of ciprofloxacin derivatives 4a-j.

**Fig 8:**
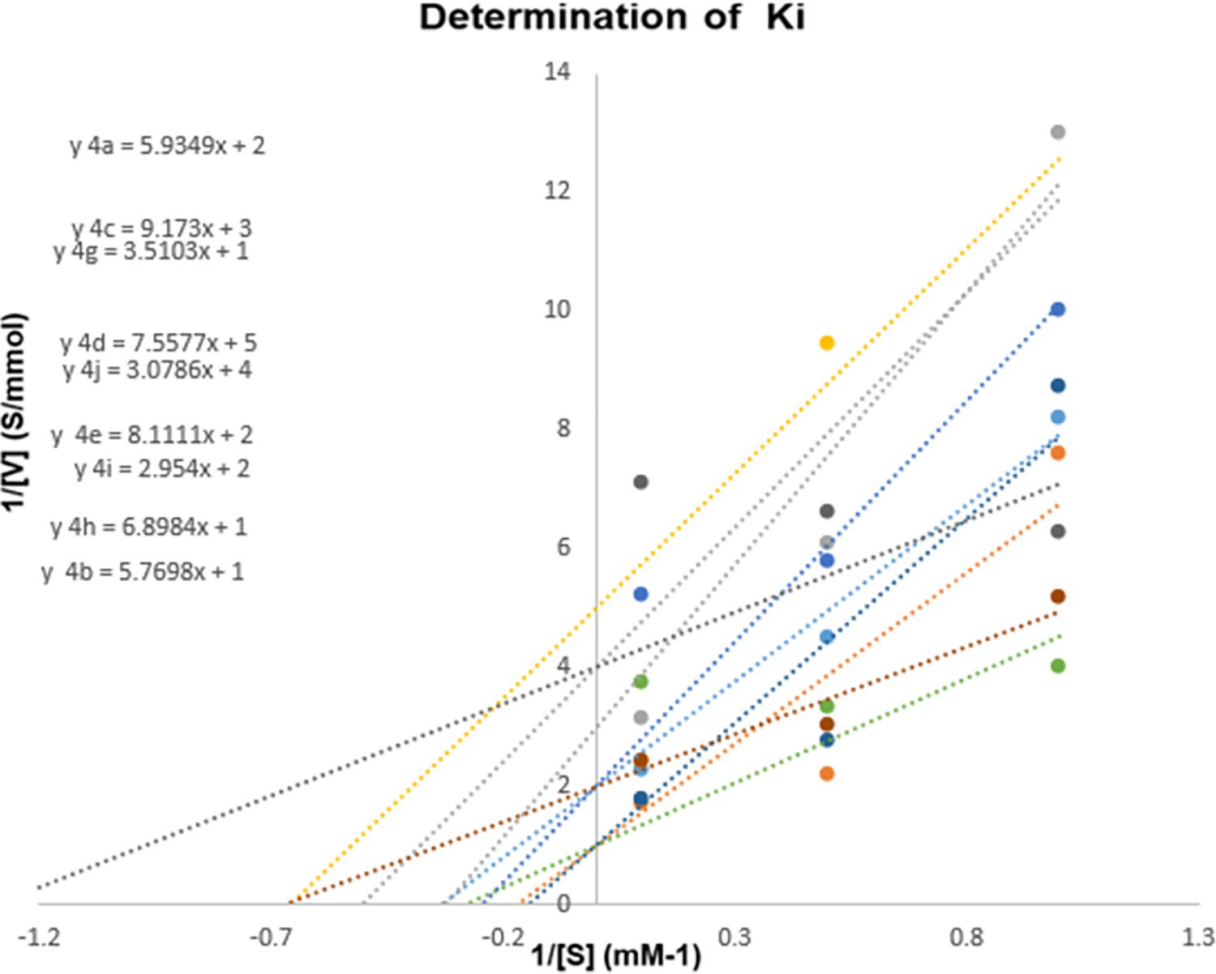
Determination of inhibition type and kinetic parameter.

The affinity of *N*-4-piperazinyl ciprofloxacin-aniline derivatives with industrially important serine protease was also investigated. *N*-alkylated ciprofloxacin derivatives (4a-j) showed an effective inhibition profile against serine proteases. Results (Figs 5–7) clearly showed that the activity of bacterial serine protease (100% inhibition) was dramatically inhibited by methyl 1-cyclopropyl-6-fluoro-4-oxo-7-(4-(2-oxo-2-(phenylamino)ethyl)piperazin-1-yl)-1,4-dihydro-quinoline-3-carboxylate **4a** at 96 minutes with half-life of 22.50 minutes. However, at 0 min. compound **4a** displayed 68% inhibition which gradually increased to 100% by increasing time from 24 to 96 minutes with *K*_*i*_ value 0.1±0.00μM (Fig 8). Promising inhibitory activity of this compound might be due to the presence of substituent free anilide ring which gave significant results as compared to the substituted derivatives. Results showed that the relative activity of enzyme and its inhibition were time-dependent [24–26]. Furthermore, half-life of compound **4b** was found to be 20.8 minutes which showed maximum inhibition potential of 88% at 96 minutes. The results revealed that presence of electron-donating methyl groups at 2,4-positions of the anilide ring of compound **4b** increased the protease inhibition activity as compared to **4a**. Similarly, methyl 1-cyclopropyl-6-fluoro-7-(4-(2-((2-methoxyphenyl)amino)-2-oxoethyl)piperazin-1-yl)-4-oxo-1,4-dihydroquinoline-3-carboxylate **4c** enhanced the protease inhibition activity at initial stage (40% inhibition at 0 min.) but later on, displayed maximum inhibition (90%) (at 96 minutes with half-life 21.3 minutes) which could be associated to methoxy group present at *ortho* position of the anilide ring of **4c**. It is interesting to report that besides these electron donating groups (OMe, Me), the presence of the fluoro group at anilide ring increased the half-life to a greater extent as compound **4g** displaying maximum half-life of 75.32 minutes with modest inhibitory activity (up to 65% at 96 minutes). While, ciprofloxacin derivatives **4e** and **4i** having chloro functionality at 4- and 3,4-postions of the anilide ring exhibited 70% and 41% inhibition potential at 96 minutes, respectively. As compared to the parent drug ciprofloxacin (15% inhibition at 0 min. and 55% at 96 min.), compound **4j** proved to be the least active one (16% inhibition at 0 min. and 29% at 96 min.) having chloro substituent at *ortho* position of the anilide ring while compounds **4b**, **4g** and **4h** were found to be competitive inhibitors. It is concluded that ciprofloxacin derivatives **4a-j** have a positive contribution to inhibit the catalytic function of protease enzyme and anilide ring played a pivotal part in this regard. Ciprofloxacin derivative **4a** with unsubstituted anilide ring significantly inhibited the catalytic activity of serine protease as compared to the other synthesized derivatives having alkyl, alkoxy and halogen groups at anilide ring. Finally molecular docking was performed to further investigate the ciprofloxacin derivative **4a**-enzyme binding interaction detail.

### *In-silico* molecular modelling

The induced fit docking was performed to further investigate the binding affinity, binding mode, and inhibitory mechanism of compound **4a** for serine protease. It is a sophisticated approach to simulate flexibility of binding pocket upon ligand binding, thereby predicting the accurate binding mode and complexation of ligand with receptor. Herein, IFD highlighted ciprofloxacin and compound **4a** conformed to energetically favorable poses with negative binding free energy (ΔG_bind_), reflecting the affinity of these ligands towards protease (Table 3). The binding affinity of ciprofloxacin served as a standard threshold. Ciprofloxacin was found to dock with -6.372 kcal/mol of Glide score, and induced fit structural optimization resulting into IFD complexation with -376.7 kcal/mol of IFD score. The Prime/MM-GBSA further approximated the thermodynamics of IFD complex, and indicated the binding of ciprofloxacin with -84.23 kcal/mol of ΔG_bind_. Intriguingly, compound **4a** was found to significantly exceed the standard’s threshold. It was found to dock with -10.03 kcal/mol of Glide score, and induced fit structural optimization producing the IFD complex with -379.6 kcal/mol of IFD score. The Prime/MM-GBSA approximated the thermodynamics of compound **4a** IFD complex with -107.62 of binding free energy (ΔG_bind_), thus highlighting the improved or superior binding affinity of compound **4a** as compared to ciprofloxacin.

**Table 3:** Parameters for induced fit docking and Prime/MM-GBSA simulation of test ligands at binding pocket of protease.

Ligand	Glide Score (Kcal/mol)	IFD Score (Kcal/mol)	Prime MM/GBSA ΔG_bind_ (Kcal/mol)	Interacting residues	Interaction types
Ciprofloxacin	-6.372	-376.7	-84.23	ASP36, SER97, LEU125, ILE70	Hydrogen bonding
Compound **4a**	-10.03	-379.6	-107.62	SER97, ILE70, HIP122, THR168	Hydrogen bonding, π-cation, π – π stacking

**Fig 9:**
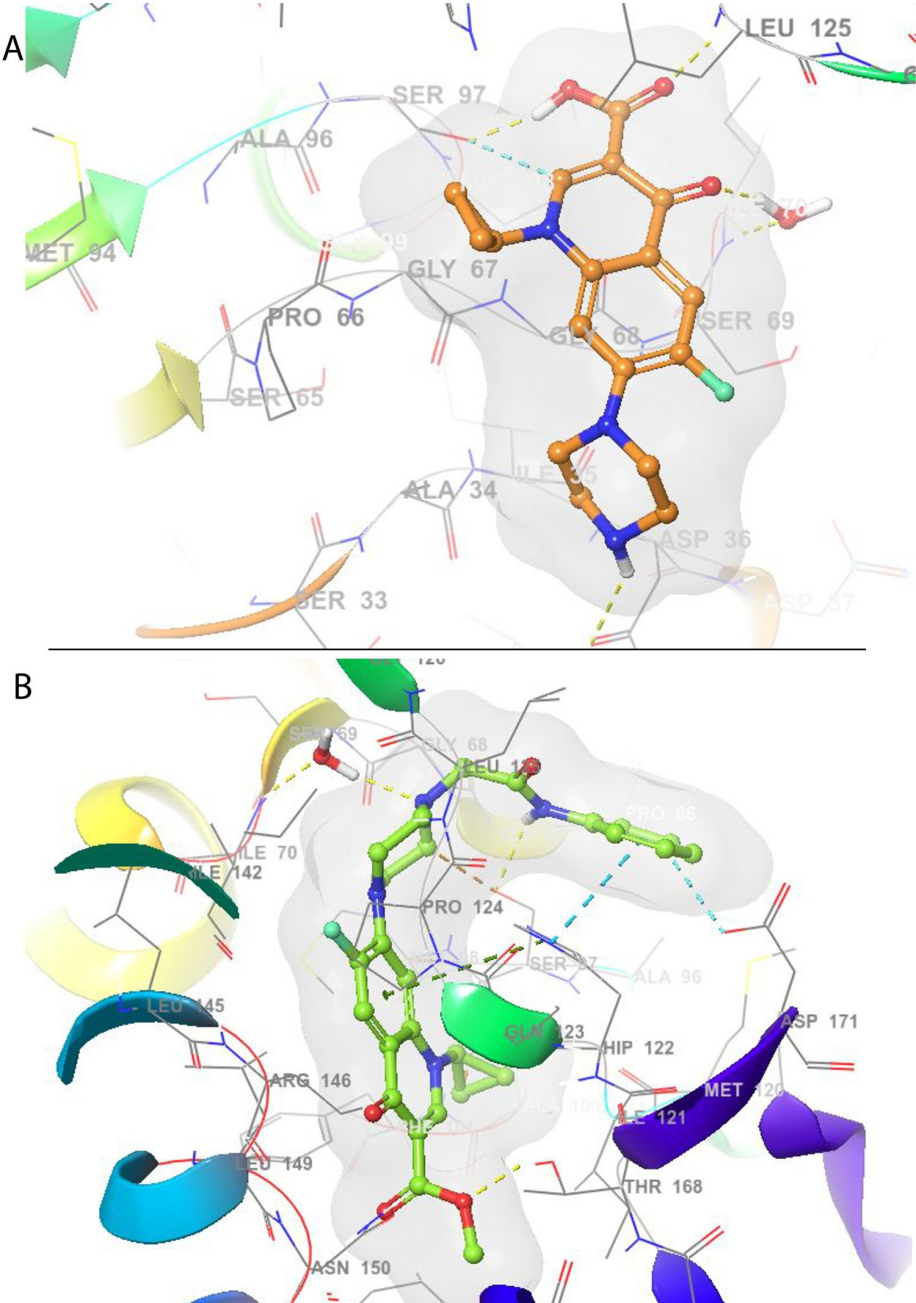
Conformational analysis of ligands at the active site of protease. Simulated best binding mode of ciprofloxacin (A) and compound 4a (B) Induced Fit docked into binding pocket.

The catalytic activity of protease is regulated by the SER97-HIS122-ASP171 catalytic triad residing deep within the enzyme. The conformational analysis showed that ciprofloxacin and compound **4a** conformed within the binding pocket for efficient access to the active sites establishing diverse interactions with vital residues (Fig 9). Though enlarged axial pore was observed upon ciprofloxacin binding, yet efficiently deep penetration into catalytic site was necessary to inhibit the enzymatic activity. Interestingly, compound **4a** efficiently blocked the axial pore to inhibit the substrate entry, and resulted into extended and distorted conformation of protease. Moreover, on deep penetration towards catalytic site, it established vital interactions to disrupt the catalytic activity of protease, and thus exhibited superior binding affinity and inhibitory potential.

**Fig 10:**
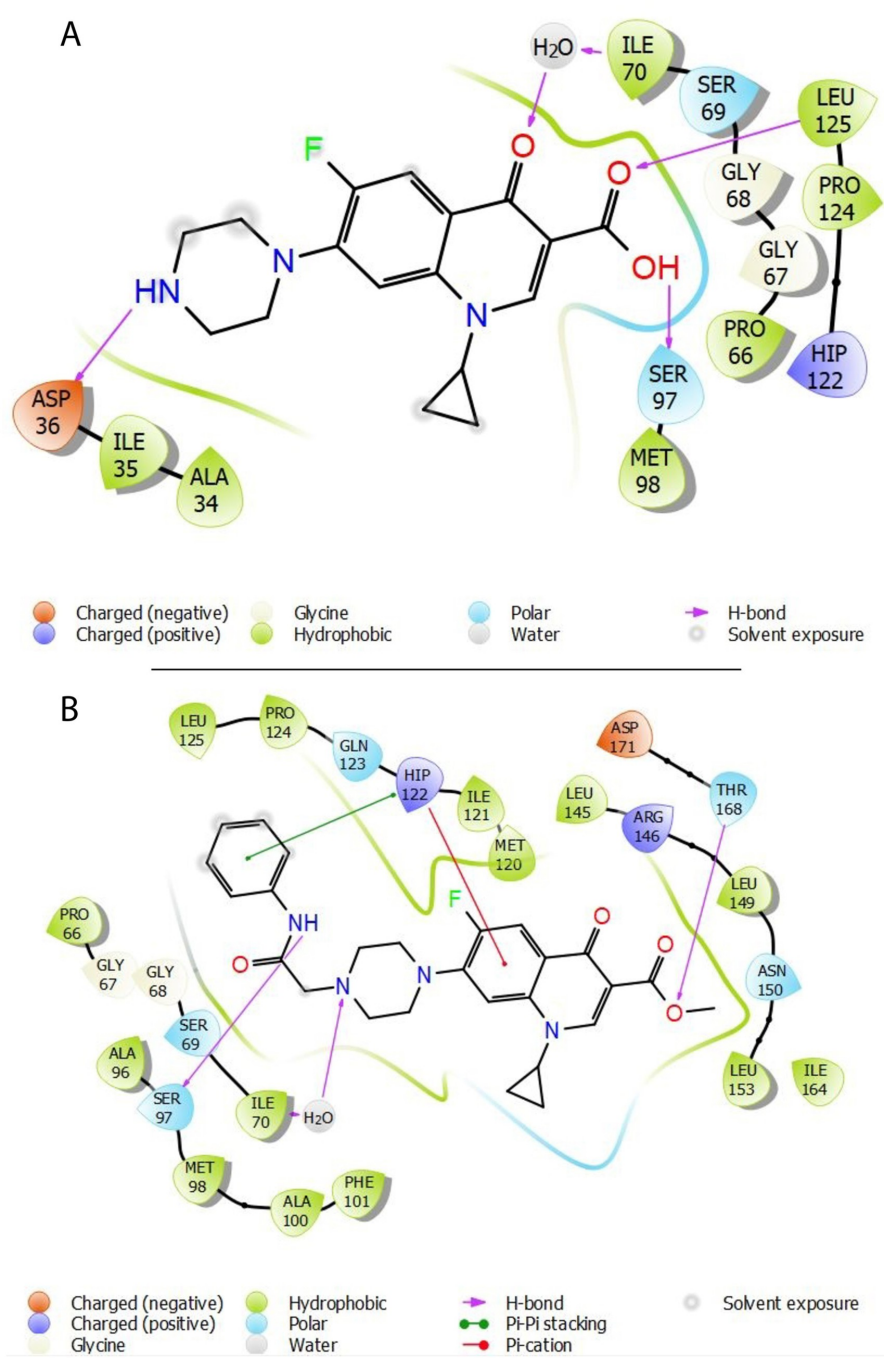
Ligands interaction profile within the active site of protease. Two dimensional (2D) illustration of ciprofloxacin (A) and compound 4a (B) interacting with active site residues.

Ciprofloxacin established its conformation mainly via hydrogen bonding. Its 3-carboxylate moiety formed a strong hydrogen bond with SER97, which is critical for the catalytic activity of serine protease, and may justify the penetration of ciprofloxacin towards catalytic triad (Fig 10). Moreover, carbonyl group of this moiety supported the conformation by hydrogen contact with hydrophobic LEU125 residue. The 4-keto and piperazinyl alkylamine groups further formed the hydrogen bond with ILE70 and ASP36, respectively. Collectively, these interactions highlighted the potential ciprofloxacin sites, which may be vital for its potency towards protease. Interestingly, compound **4a** shared the majority of its conserved and vital interactions within the binding pocket of protease. Compound **4a** established its conformation by diverse hydrophobic and hydrophilic interactions. It was noteworthy that anilide substitution at piperazinyl secondary amine significantly changed the interaction profile of ciprofloxacin, and resulted into more diverse interactions via this moiety. The secondary amine of this phenylamine substitution formed a strong hydrogen bond with SER97, and disrupted the catalytic triad of serine protease. Moreover, phenyl ring of this moiety along with cycloalkene benzene ring of quinoline established the pi-pi stacking and pi-cation interactions with HIS122 at the catalytic triad, respectively. These hydrophobic interactions formed the extended conformation of protease by increasing the distance between SER97 and HIS122, this may have severely inhibited its catalytic activity by corroborating the superior catalytic disruption as compared to the ciprofloxacin. Furthermore, 3-carboxylate moiety and *N*-4 piperazinyl tertiary amine established the hydrogen interactions with THR168 and ILE70, respectively. These interactions further disrupted the catalysis via blockade of substrate entry through axial pore. Therefore, these structural insights may provide plausible insights to explain that compound **4a** possesses superior binding affinity and inhibitory potential for protease.

## Conclusions

In the present work, enzyme inhibition potential of designed series of *N*-alkylated ciprofloxacin hybrids exhibited satisfactory results by displaying the maximum inhibition potential of serine protease. Among the title compounds, **4a** showed maximum inhibition (100%) at 96 minutes with a half-life of 22.50 minutes. The IFD/MM-GBSA studies highlighted the binding mode of **4a** for protease inhibition and indicated improved binding affinity with– 107.62 kcal/mol of ΔG_bind_. In addition to this, antibacterial study of the synthesized derivatives revealed that the compounds **4e** and **4g** were the most potent against *Bacillus subtilis* with ZI values 40 mm and 37 mm, respectively. However, in case of *Escherichia coli*, compounds **4a** and **4i** displayed maximum ZIs values i.e. 38 mm and 46 mm, respectively. These results highlight the significance of *N*-alkylated ciprofloxacin derivatives for further *in vivo* evaluation and would be helpful for designing pharmacologically important safe and potent drugs in future.
